# Constant light disrupts biological rhythms and worsens sleep quality but does not elevate blood pressure in female rats

**DOI:** 10.1038/s41440-026-02579-8

**Published:** 2026-02-19

**Authors:** Lubos Molcan, Hana Mauer Sutovska, Michal Zeman

**Affiliations:** Department of Animal Physiology and Ethology, Faculty of Natural Sciences, Comenius University in Bratislava, Bratislava, Slovakia

**Keywords:** Constant light, Blood pressure, Electroencephalography, Female rats, Morning hypertension

## Abstract

Constant light (LL) disrupts biological rhythms, although more data are available on circadian than on ultradian rhythms. LL has been linked to elevated blood pressure (BP), although most evidence comes from tail-cuff plethysmography in males. However, in nocturnal animals, LL should suppress activity, increase sleep, and lower BP. Therefore, the aim of this study is to provide a comprehensive analysis of the impact of LL on (1) cardiovascular parameters and sleep and (2) circadian and ultradian variability in female rats. We used telemetry for continuous monitoring of heart rate (HR), BP, and locomotor and sleep-wake activity in female rats exposed to LL for four weeks. LL progressively reduced basal systolic BP and HR and weakened the strength of circadian rhythms. Moreover, the loss of daily variability enhanced the acute cardiovascular response. Spectral analysis revealed disrupted ultradian rhythms, with HR power shifting from longer (~7–9 h) to shorter (~1–3 h) periods and locomotor activity showing a parallel decline, including a complete loss of 7–9 h rhythms by week 4. HR variability and baroreflex analysis showed parasympathetic dominance under LL. Sleep analysis revealed significant sleep disruption, characterised by altered distribution of sleep–wake states, reduced non-REM sleep during the light phase, increased fragmentation, and a complete loss of circadian organisation. LL reduced BP in female rats despite leaving total sleep duration largely unchanged, while markedly disrupting cardiovascular circadian and ultradian variability and sleep architecture. These findings suggest that LL-induced chronodisruption imposes a maladaptive physiological load also in nocturnal rodents.

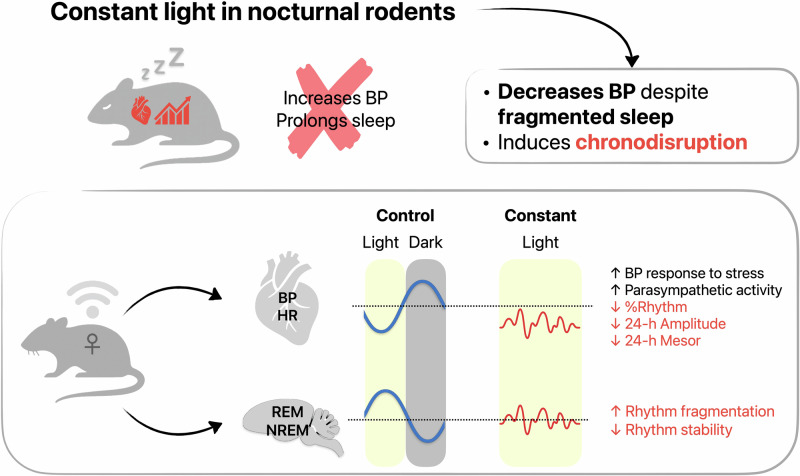

## Introduction

Biological processes exhibit endogenous oscillations that reflect the functionality and adaptability of physiological systems [1]. The most thoroughly studied processes are the circadian rhythms (approximately 24 h), governed by the suprachiasmatic nuclei (SCN) of the hypothalamus. The SCN entrains internal rhythms with the light-dark cycle, ensuring proper timing of a broad range of biological functions [2]. Disruptions of circadian rhythmicity caused by chronic exposure to shift work, jet lag, or light at night are associated with the development of metabolic [3, 4], cardiovascular [5, 6], and neurological [7, 8] disorders.

Beyond circadian oscillations, the body also exhibits the whole spectrum of shorter-period rhythms, known as ultradian oscillations. These are involved in the control of behaviour, neuroendocrine activity, and autonomic functions [1, 9, 10]. Experimental data suggest that these rhythms may operate at least partially independently of the SCN [11], persisting even after SCN lesions or during constant light (LL) exposure [12]. Thus, LL exposure research is valuable for investigating the consequences of disrupted circadian organisation and enables the study of ultradian variability without the effects of SCN. Whereas harmonic ultradian rhythms remained stable under LL, the circadian variability in heart rate (HR) and blood pressure (BP) was disrupted [13].

Experimental rodent studies, based on tail-cuff plethysmography, have reported elevated BP and adverse cardiovascular effects in rats kept under LL [14, 15]. However, this method can confound results by increasing stress and animal reactivity [16, 17]. Therefore, in this study, we employed both tail-cuff plethysmography and continuous telemetry to evaluate the effects of LL on cardiovascular parameters. Furthermore, we investigated how LL-induced chronodisruption affects restorative sleep architecture and its relationship to cardiovascular parameters. This question remains unexplored in nocturnal animals that are expected to rest more under LL. Since previous research has focused mostly on males, in this study, we provide a comprehensive analysis of the impact of LL on circadian and ultradian variability of cardiovascular parameters and sleep in female rats.

## Materials and methods

### Animals

We used adult female Wistar rats (*n* = 10; 10–12 months old; 307 ± 3 g) from the breeding facility at the Institute of Experimental Pharmacology and Toxicology, Slovak Academy of Sciences (Dobrá Voda, Slovak Republic). The animals were housed individually in transparent plastic cages, with food and water provided ad libitum. Environmental conditions, including room temperature (21–23 °C), humidity (50–70%), and light intensity (light: ~120 lx at cage level; dark: 0 lx; ZT0 = 6:00, the beginning of the light phase), were continuously monitored and recorded using dataloggers. The experiment was approved by the Ethical Committee for the Care and Use of Laboratory Animals at Comenius University in Bratislava and the State Veterinary Authority of the Slovak Republic. All procedures complied with the ARRIVE guidelines, the Guide for the Care and Use of Laboratory Animals, and EU Directive 2010/63/EU on animal experiments.

### Study design

Before the experiment, the rats were implanted with pressure (*n* = 4) or electroencephalography (EEG; *n* = 6) telemetry sensors. Following sensor implantation, the rats were housed individually in transparent plastic cages placed on telemetry receivers under standard 12 h light and 12 h dark cycles (LD). Subsequently, the animals were exposed to four weeks of constant light (LL; 500–550 lx at cage level). BP, HR, EEG, electromyography, and locomotor activity were continuously recorded during the control LD week (days –7 to 0) and on a weekly basis during LL (Supplementary Fig. S1). To study stress response, the home cage shaking test (45 s) was performed during the control LD regime (day –3 at ZT13; day 0 at ZT03) and in the fourth LL week (day 29 at the original ZT03).

### Telemetry

Telemetry sensors were implanted into anaesthetised rats (induction: 4% isoflurane in 100% oxygen; maintenance: 1.5–2% isoflurane in 100% oxygen; flow 0.5 L/min). For BP recording, TA11PA-C40 sensors (DSI, USA) were used. The rat was positioned in dorsal recumbency, and a sterile field was established. A 4–5 cm midline abdominal incision was made to expose the aorta, which was carefully separated from the vena cava. Occlusion sutures were placed cranial to the iliac bifurcation, and a 22-gauge needle was used to introduce the telemetry catheter into the aorta, advancing it cranially. To prevent leakage, the catheter was secured in the aorta with tissue glue (Histoacryl®, B. Braun). The transmitter body with battery was placed in the abdominal cavity and sutured to the abdominal muscle wall.

For EEG recording, the rat was immobilised in sternal recumbency, and a dorsal neck incision was made. The skull was exposed, the periosteum removed, and perforations created at predefined coordinates. The negative lead of the EEG HD-S02 transmitter (DSI, USA) was placed 2.0 mm anterior, 1.5 mm lateral to bregma, and the positive lead was placed 7.0 mm posterior, 1.5 mm lateral to bregma contralaterally. The screw attachment method secured the electrodes, which were wrapped around partially inserted screws, and then fully advanced for direct contact with the dura. Leads were fixed and embedded in dental acrylic for stability and isolation. For postural muscle electromyography, biopotential leads were implanted in the dorsal neck muscles (2–3 mm apart), secured with sutures to prevent movement, and positioned to lie flat under the skin to avoid tension or necrosis. The transmitter body containing the battery was placed intraperitoneally in accordance with the manufacturer’s surgical manual.

After surgery, ampicillin (50 mg/kg, subcutaneously) and tramadol (15 mg/kg, subcutaneously) were administered, and the animals were allowed to recover for one week.

### Tail-cuff plethysmography

Systolic BP was also measured weekly using a non-invasive tail-cuff plethysmography system (PowerLab 4/30, ADInstruments). Before the measurement session, the rats were habituated to the procedure to reduce stress-related variability. Measurements for each week were conducted on the following Monday, e.g., data corresponding to the LD week were collected on the Monday of LL1 (Supplementary Fig. S1). Rats were pre-warmed to approximately 38 °C before each session. For each rat, multiple measurements were performed within approximately five minutes. All sessions were conducted consistently during ZT01–ZT03. To minimise variability, the same people made the measurements each week, and rats were measured in the same order throughout the study.

### Data acquisition and processing

Aortic BP was recorded via telemetry using the Dataquest A.R.T. version 4.36 system (DSI, USA) at a 500 Hz sampling frequency. From the high-resolution pressure waveform, we extracted HR and systolic BP. Locomotor activity was simultaneously recorded via the secondary channel of the telemetry transmitter. Due to animal handling on experimental days -3 and 0, LD-week analyses were based on days –7 to –4. HR, BP, and activity data were exported as minute and hourly averages for further study.

EEG and electromyogram signals were recorded using Ponemah version 6.51 (DSI, USA) and later analysed in NeuroScore version 3.4 (DSI, USA) using the Rodent Sleep Scoring 2 algorithm. From the original waveform signals (cortical EEG, theta-filtered EEG, and neck electromyogram sampled at 500 Hz; activity sampled at 1 Hz), we generated hourly epochs of wake, active wake, paradoxical sleep (rapid eye movement, REM), and slow-wave sleep (non-REM).

Given the known effects of LL on period lengthening and rhythm loss (Aschoff’s rules), L/D subdivision under LL would not reflect actual physiological phases [18]. Therefore, we analysed BP, HR, locomotor activity, and sleep-wake states separately for L and D phases in LD week. In contrast, each LL week was analysed as a whole, without subdivision into light and dark phases.

To study the acute physiological and behavioural response to a short-term shaking stressor. We analysed three outcome measures for HR, systolic BP, and locomotor activity: (1) the area under the curve (AUC) over the 60 min poststimulus; (2) the maximal value reached during the first 10 min; and (3) the mean value during the same 10-min, poststimulus period. All results were normalised to each rat’s individual 60-min prestimulus baseline value. To visualise and perform statistical calculations, we used 1-min averages.

#### Circadian and ultradian variability

For cardiovascular and locomotor activity parameters, which were continuously and evenly sampled, we used a complementary parametric approach via Chronos-Fit version 1.06 software [19]. This software allows the decomposition of the signal into multiple harmonic components and quantification of rhythm characteristics, including %Rhythm (explained variance), the amplitude of the 24-h component, mesor (baseline mean level), and F-statistic (strength of rhythmicity), and calculates periodogram in the broad spectral range. To better capture the temporal rhythmicity dynamics, we compared the average spectral power of Lomb-Scargle periodogram analysis within seven consecutive ultradian time intervals (1–3 h, 3–5 h, 5–7 h, 7–9 h, 9–11 h, 11–13 h, and 13–20 h) for each rat and parameter. We analysed the first 50 most significant periods in each ultradian time interval.

#### Intradaily variability and interdaily stability

To evaluate circadian and ultradian rhythmicity in physiological and behavioural outputs, we applied complementary analytical approaches depending on the nature of the variables. EEG vigilance states (wake, active wake, REM, and non-REM) were analysed using non-parametric rhythm metrics suitable for event-like or time-in-state data. Specifically, we used intradaily variability (IV; fragmentation of the rhythm within a 24-h period), quantifying the frequency and intensity of transitions between vigilance states across the 24-h period. IV was calculated as the ratio of the mean squared successive differences to the overall variance [20]. Low IV values correspond to consolidated activity or rest phases, while higher values indicate frequent transitions between active and inactive states, reflecting a more fragmented rhythm.

To quantify the degree to which daily activity patterns were replicated across consecutive days, we used interdaily stability (IS; rhythm stability). It was computed as the normalised variance of hourly means across days [20]. Higher IS values indicate a regular and stable rhythm, whereas lower values reflect weaker or more irregular patterns.

In addition, 24-h peak spectral power was estimated using the Lomb-Scargle periodogram, a method suitable for unevenly sampled time series [20]. The peak amplitude was defined as the highest spectral power in the 23–25 h frequency range, interpreted as the strength of the circadian component.

#### Time- and frequency-domain analysis of HRV

HRV (heart rate variability) was analysed from 5-min segments. In the time domain, standard measures were computed. SDNN was calculated as the standard deviation of all normal-to-normal (NN) intervals, reflecting overall HRV. RMSSD was derived as the square root of the mean of the squared differences between successive NN intervals, providing an estimate of short-term HRV mediated primarily by parasympathetic activity.

In the frequency domain, HRV parameters were derived from inter-beat intervals (IBI), detected from systolic BP peaks, using the Lomb–Scargle periodogram, which allows spectral analysis of unevenly sampled time series. IBI intervals were first converted to milliseconds (ms) and analysed within a defined frequency range that included both low-frequency (LF, 0.25–0.75 Hz) and high-frequency (HF, 0.75–2.5 Hz) bands. LF and HF power (ms²) were obtained as the integrated spectral power within the respective bands. LF (nu) and HF (nu) were calculated as the proportion of LF or HF power relative to the total power in both bands. The LF/HF ratio reflects the balance between sympathetic and parasympathetic activity.

#### Spontaneous baroreflex sensitivity

Baroreflex sensitivity (BRS) was calculated using the sequence method based on three-point sequences of systolic BP and inter-beat intervals (IBI). The algorithm identified all sequences of three consecutive systolic BP and IBI values in which both variables exhibited a consistent increasing or decreasing trend. A sequence was considered valid if the absolute change between adjacent points exceeded predefined thresholds (≥1 mmHg for systolic BP and ≥5 ms for IBI). For each valid sequence, a linear regression was performed with IBI (ms) as the dependent variable and systolic BP (mmHg) as the independent variable. The slope of the regression line was taken as the index of BRS (ms/mmHg). The final BRS value for the segment was calculated as the mean of all regression slopes from valid sequences. Sequences with opposing trends or changes below threshold values were excluded from the analysis.

### Statistical analysis

Differences between experimental conditions were assessed using linear mixed-effects models, that included the week as a fixed effect and rat as a random intercept to account for repeated measures. The core assumption of normality of model residuals was evaluated using the Shapiro-Wilk test. If the assumption was met (*p*≥0.05), the significance of fixed effects was determined using a Type III analysis of variance, followed by appropriately adjusted post-hoc comparisons. For direct comparisons (dark vs. light phase), paired t-tests were applied. If the assumption was violated (*p* < 0.05), a robust non-parametric approach was employed. For paired comparisons (dark vs. light phase), the Wilcoxon signed-rank test was used. For multiple group comparisons, the Friedman test was used when datasets were complete; in cases with missing values, a linear mixed-effects model on rank-transformed data was applied. This latter method was chosen for its robustness, as it correctly handles potentially unbalanced datasets due to missing values while maintaining a non-parametric analysis framework.

Linear and exponential regression models were used to assess temporal trends across weeks. A *p* value of <0.05 was considered statistically significant for all tests.

All statistical analyses and visualisations were performed in R software (v 4.4.2; R Core Team). Data were processed and reshaped using the packages dplyr, readr, tidyr, and tidyverse. Statistical modelling was conducted using afex, Companion to Applied Regression, emmeans, lme4, lmerTest, pbkrtest, and rstatix. Visualisations and figure layouts were generated using cowplot, ggplot2, ggpubr, ggsci, grid, gridExtra, patchwork, and scales. For time-series and rhythmicity analyses, we used the Lomb and broom packages.

## Results

### Cardiovascular and locomotor activity changes

**Fig. 1:**
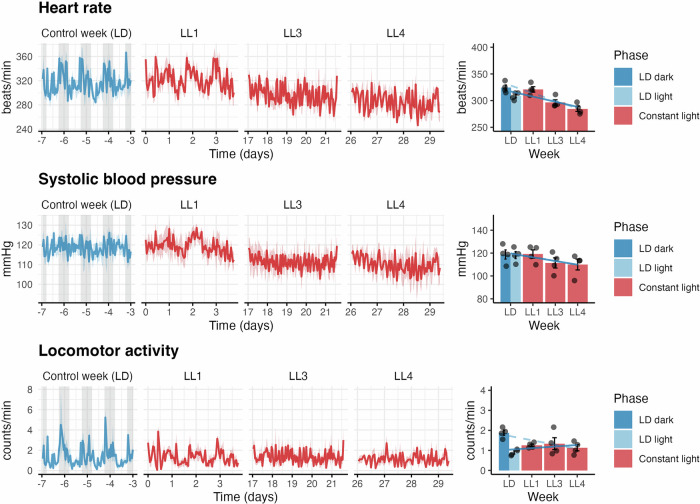
Cardiovascular and behavioural responses to constant light exposure. Time series and weekly averages of heart rate, systolic blood pressure, and locomotor activity during a light-dark (LD) week (control) and constant light weeks (LL1, LL3, LL4). The grey areas in the LD panels represent the dark phases. Weekly averages are presented as means ± SEM, with individual animals shown as dots

Measured by telemetry, during the control LD week, HR and locomotor activity were significantly higher in the dark phase (*p* = 0.042 and *p* = 0.001, respectively). At the same time, systolic BP did not differ between phases. Exposure to LL led to progressive changes in HR, systolic BP, and locomotor activity, with the pattern and statistical significance depending on the reference phase used for comparison (Fig. 1). For HR, the week had a significant effect (*p* < 0.001). Compared to the LD-dark, HR significantly decreased in LL3 and LL4 (both *p* < 0.001), with a decreasing trend (β = –14.345, *p* < 0.001, R^2^ = 0.736). A similar pattern was observed when comparing LL data to LD-light, with a significant regression (β = –9.605, *p* = 0.004, R^2^ = 0.466) and reduction in LL4 (*p* = 0.001). Systolic BP also showed an effect of the week (*p* = 0.003). BP was lower in LL3 and LL4 compared to both LD-dark (*p* = 0.027 and *p* = 0.010) and LD-light (*p* = 0.046 and *p* = 0.018), with marginal regression significance (light: β = –3.250, *p* = 0.081, R^2^ = 0.202; dark: β = –3.409, *p* = 0.079, R^2^ = 0.204). Locomotor activity was reduced compared with LD-dark (*p* = 0.047), with a significant effect in LL4 (*p* = 0.045, Tukey post-hoc), but no changes compared with LD-light. The decrease was found only for the LD-dark comparison (β = –0.216, *p* = 0.022, R^2^ = 0.323).

Exposure to LL induced significant alterations in cardiovascular autonomic control, leading to a pronounced shift towards parasympathetic dominance compared to the baseline LD condition (Table 1, Supplementary Table S1). This shift was evidenced by a significant decrease in sympathetic activity (LF nu) and a corresponding increase in parasympathetic activity (HF nu), particularly during LL3 (*p* < 0.01) and LL4 (*p* < 0.01). Consequently, the LF/HF ratio was significantly lower during week LL3 (*p* < 0.01) and LL4 (*p* < 0.01) compared to baseline. In line with these changes, indicators of vagal tone such as RMSSD and HF power, as well as overall HR variability (SDNN), were significantly increased following extended LL exposure. BRS was also considerably affected throughout the experiment (Friedman test, *p* = 0.017), showing a clear trend of increasing sensitivity. In contrast, no statistically significant differences were observed between the dark and light phases for any of the monitored parameters during the baseline LD week (Table 1; Supplementary Table S1).

**Table 1:** Effect of constant light exposure on heart rate variability (HRV) parameters and baroreflex sensitivity (BRS)

Parameter	LD dark	LD light	LL1	LL3	LL4
SDNN (ms)	12.65 ± 0.75	13.70 ± 0.76	13.17 ± 0.72	13.86 ± 0.70	13.36 ± 0.43
RMSSD (ms)	4.41 ± 0.12	4.80 ± 0.32	4.90 ± 0.52	6.14 ± 1.06	6.03 ± 0.98
LF power (ms²)	1.44 ± 0.07	1.34 ± 0.12	1.47 ± 0.17	1.63 ± 0.30	1.64 ± 0.34
HF power (ms²)	2.14 ± 0.12	2.19 ± 0.08	2.31 ± 0.30	3.81 ± 0.96	4.02 ± 1.12
LF nu	0.440 ± 0.007	0.420 ± 0.012	0.435 ±± 0.010	0.369 ± 0.021**	0.352 ± 0.016**†
HF nu	0.560 ± 0.007	0.580 ± 0.012	0.565 ± 0.010	0.631 ± 0.021**	0.648 ± 0.016**†
LF/HF	0.881 ± 0.029	0.821 ± 0.042	0.865 ± 0.034	0.664 ± 0.067**	0.613 ± 0.048**†
BRS (ms/mmHg)	3.91 ±± 0.14	3.78 ± 0.19	3.76 ± 0.21	5.11 ± 0.81	5.45 ± 1.19

Data are presented as mean ± SEM

Statistical analysis was performed using linear mixed-effects models or paired *t* tests with Tukey correction for multiple comparisons. For BRS, a non-parametric Friedman test was applied (overall *p* = 0.017). Units for low-frequency (LF) and high-frequency (HF) power are ×10⁻⁴. LD, 12 h light / 12 h dark; LL1, constant light week 1; LL3, constant light week 3; LL1, constant light week 4; SDNN, standard deviation of all normal-to-normal (NN) intervals; RMSSD, square root of the mean of the squared differences between successive NN intervals; LF nu, LF power of HRV expressed in normal units; HF nu, HF power of HRV expressed in normal units; ms, milliseconds, **p < 0.01 vs. LD dark; †*p* < 0.05 vs. LL1

Systolic BP measured by tail-cuff plethysmography showed no consistent pattern over the studied period of four weeks. Individual responses varied: three rats showed increases; two rats showed decreases; three rats showed no change (Supplementary Fig. S2A). Group-level ANOVA revealed no effect of the week (*p* = 0.242). Bonferroni post-hoc tests confirmed non-significance, indicating stable group BP across weeks (Supplementary Fig. S2B).

### Circadian rhythms in cardiovascular and locomotor activity

**Fig. 2:**
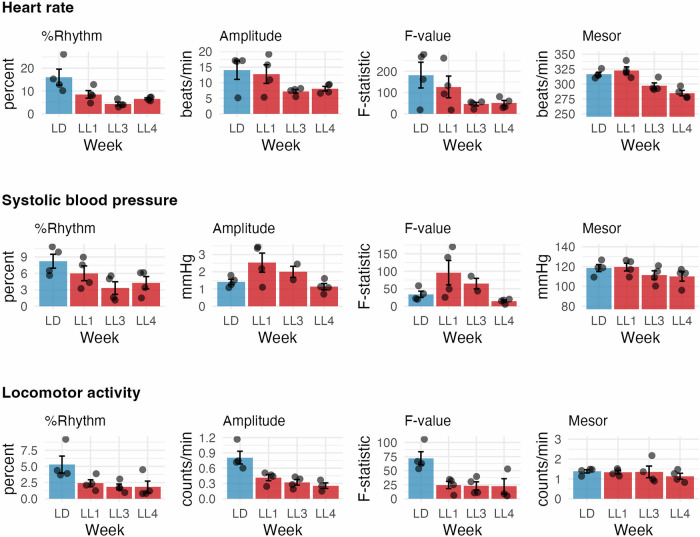
Circadian rhythm parameters of heart rate, systolic blood pressure, and locomotor activity in female rats under light-dark (LD) and constant light (LL) conditions. Mean values ± SEM for circadian rhythm parameters derived from cosinor analysis across each week: rhythm percentage (%Rhythm), amplitude, F-value (strength of rhythm), and mesor (rhythm-adjusted mean) are shown. LD represents the control week under a light-dark cycle; LL1, LL3, and LL4 represent weeks 1, 3, and 4, respectively, of LL. Bars represent group means ± SEM; dots indicate individual rats

For HR (Fig. 2), %Rhythm significantly declined across LL weeks (*p* = 0.038). Linear regression confirmed this trend (β = –3.272; R² = 0.426, *p* = 0.006). The mesor dropped (*p* < 0.001) from 316 ± 3 bpm (LD) to 285 ± 5 bpm (LL4), showing a clear LL-duration dependency. Amplitude showed a marginal decline (*p* = 0.075), while the F-value decreased with borderline significance (*p* = 0.058). For systolic BP, %Rhythm declined (β = –1.447; R² = 0.329, *p* = 0.020), and mesor was significantly lower (*p* = 0.002). Amplitude and F-value showed no significant changes. For locomotor activity, %Rhythm, F-value, and amplitude significantly decreased (*p* < 0.001, *p* < 0.001, *p* = 0.004), with all LL weeks lower than LD. All showed significant linear trends (*p* = 0.017, *p* = 0.011, *p* < 0.001). Mesor remained unchanged (*p* = 0.664).

### Ultradian rhythms in cardiovascular and locomotor activity

**Fig. 3:**
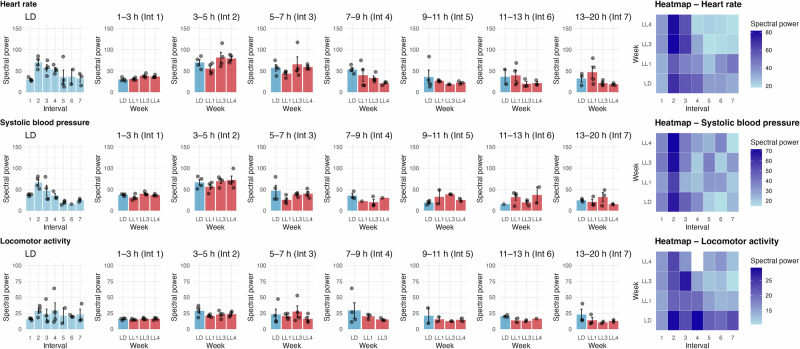
Ultradian rhythmicity of heart rate, systolic blood pressure, and locomotor activity in female rats across light–dark (LD) and constant light conditions. Spectral power was analysed in predefined ultradian frequency intervals (1–3 h, 3–5 h, 5–7 h, 7–9 h, 9–11 h, 11–13 h, and 13–20 h; labelled Int 1 to Int 7) for heart rate, systolic blood pressure, and locomotor activity across LD and constant light exposure weeks (LL1, LL3, LL4). Bars show interval-specific spectral power per week (mean ± SEM; dots represent individual rats). Right panels show corresponding heatmaps of mean spectral power per week and interval

In LD, the spectral power of all three parameters peaked between 3 and 7 h, with significant early–to–mid interval differences for HR and SBP (Fig. 3, blue columns). Under LL, ultradian variability persisted with highly variable peaks (Supplementary Fig. S3). Spectral power of HR (Fig. 3) significantly increased in the 1–3 h interval (*p* = 0.055), but was lower in the 7–9 h interval (*p* = 0.067). Linear regression confirmed trends in the 1–3 h (β = 2.820; *p* = 0.007; R^2^ = 0.415) and 7–9 h (β = –10.380; *p* = 0.015; R^2^ = 0.377) intervals. No decline in spectral power in systolic BP and locomotor activity across intervals following LL exposure was observed. However, we noted a reduction in the number of rats for which a significant period was detected within the respective frequency band. Locomotor activity power decreased in LL in several intervals (Fig. 3); in the 3–5 h interval, LL1 showed lower values than LD (*p* = 0.054). Within 7–9 h, none of the animals exhibited significant oscillation during the LL4 week. No significant differences were detected in other intervals.

### Sleep analysis

During LD, wake and active wake were higher in the dark phase (*p* < 0.001 and *p* = 0.063, respectively), whereas REM and non-REM were elevated in the light phase (*p* = 0.005 and *p* < 0.001, respectively).

**Fig. 4:**
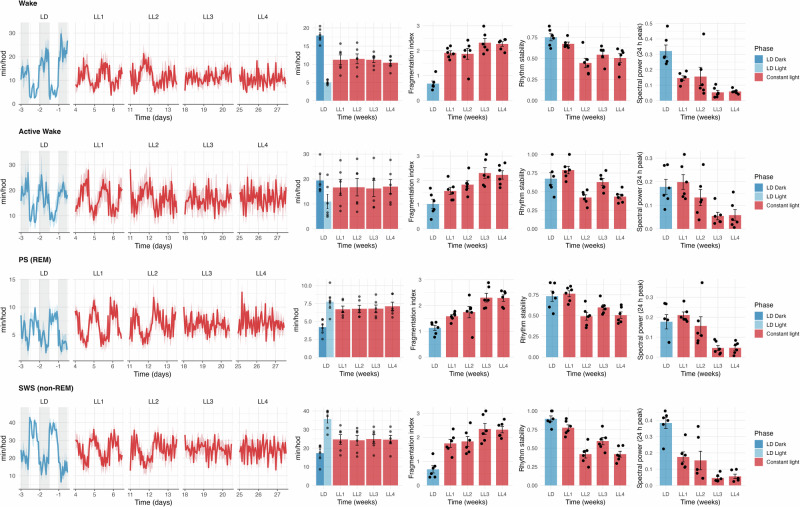
Effects of constant light (LL) exposure on sleep–wake architecture and EEG-based rhythmicity in female rats. Time series (left) and group averages (right) of wake, active wake, paradoxical sleep (PS; REM sleep), and slow-wave sleep (SWS; non-REM) across light-dark (LD; blue) cycle and LL (red). Each row shows the progression of one vigilance state across time, including average min/h, fragmentation index (IV; a measure of hour-to-hour fluctuation), rhythm stability (IS; the day-to-day consistency of the 24-h pattern), and 24 h spectral power. Bars represent mean ± SEM; dots indicate individual animals

LL exposure significantly affected wake (Fig. 4), differing from both LD-light (*p* < 0.001) and LD-dark (*p* < 0.001), but not for LD-total. Compared to the light phase, we observed a linear increasing trend (β = 1.084, *p* = 0.010, R² = 0.215) as well as an exponential trend (a = 7.354, b = 0.096, *p* = 0.027). Compared to the dark phase, both the linear (β = −1.496, *p* < 0.001, R^2^ = 0.346) and the exponential fit (a = 18.172, b = −0.132, *p* < 0.001) showed a decreasing trend. In contrast, no significant linear or exponential trend was detected when compared with LD-total.

For active wake (Fig. 4), a significant effect of the week was found for LD-light vs LL (p < 0.001), but no significant trends were detected in either linear or exponential models.

For REM (Fig. 4), LL exposure significantly affected values, differing from LD-dark (*p* < 0.001), LD-light (*p* = 0.018), and LD-total (*p* < 0.001). A significant increasing trend was evident in the LD-dark (linear: β = 0.594, *p* = 0.002, R^2^ = 0.298; exponential: a = 4.812, b = 0.088, *p* = 0.004) and LD-total (linear: β = 0.316, *p* = 0.046, R^2^ = 0.135; exponential: a = 5.711, b = 0.047, *p* = 0.047) comparison.

For non-REM (Fig. 4), significant effects of the week were found in both LD-light and LD-dark (*p* < 0.001), but not in the LD-total comparison. Regression showed a decrease in the light phase (linear: β = −2.204, *p* = 0.014, R^2^ = 0.199; exponential: a = 34.841, b = –0.090, *p* = 0.008) and a marginal increase in the dark phase (linear: β = 1.482, *p* = 0.061, R^2^ = 0.120; exponential: a = 19.272, b = 0.060, *p* = 0.080). No significant linear or exponential trend was detected when compared with LD-total.

Fragmentation index significantly increased (*p* < 0.001), while rhythm stability and spectral power peak amplitude significantly decreased (*p* < 0.001) across LL weeks in all four parameters (Fig. 4).

## Discussion

Our study revealed that exposure to LL profoundly alters multiple physiological systems in female rats. Progressive reductions in HR and systolic BP were accompanied by a shift in autonomic nervous balance and enhanced BRS. In contrast, and perhaps more surprisingly, sleep architecture was markedly disrupted, with reduced non-REM sleep during the light phase, increased REM sleep during the dark phase, and greater sleep fragmentation overall. At the same time, LL weakened circadian rhythmicity in both cardiovascular and behavioural parameters, redistributing variability into shorter ultradian intervals.

### Cardiovascular and sleep measures

Continuous telemetry revealed a significant reduction in both BP and HR under LL. Similarly, chronodisruption such as dim light at night [21, 22] and phase shifts in the LD cycle [23–25] decrease BP and HR in rats.

Consistent with these earlier observations, LL in our study increased parasympathetic activity and decreased sympathetic nervous activity and basal BP in female rats. Although LL suppresses sympathetic tone, the acute cardiovascular response to stress is paradoxically augmented [21, 26]. In our study, basal BP was reduced, yet the pressor response to shaking on the subjective day (ZT03) remained unchanged, indicating enhanced vascular reactivity (Supplementary Fig. S4). Since LL abolishes circadian rhythms, a similar response may occur during the subjective night (ZT13) due to the elimination of circadian modulation of adrenergic sensitivity. Comparable endocrine alterations have been also described, with LL abolishing the diurnal corticosterone rhythm in male rats [15, 27] and enhancing adrenocorticotrophin secretion and adrenal cortex activity in female rats [28, 29]. When SCN output is suppressed, the organism loses the ability to anticipate predictable daily stressors [30]. This may also explain reports of increased BP measured by tail-cuff plethysmography and sympathetic activity in LL-exposed animals [14, 15]. In our experiment, tail-cuff plethysmography showed high inter-individual variability and no consistent group-level change in BP. This contrasts with our telemetry data, highlighting the limitations of the tail-cuff method for long-term monitoring of LL effects. However, the increased stress response during LL may represent a maladaptive consequence of circadian disruption. In humans, exaggerated BP peaks are recognised as an early and strong predictor of stroke risk [31].

As part of the acute stress response, elevated sympathetic tone has also been reported in rats following sleep deprivation [32]. Given the close link between sleep and cardiovascular regulation, we also measured the amount and quality of sleep in nocturnal rats, not only because light is expected to promote sleep in such species [33] and wakefulness in diurnal ones [34], but also to better assess cardiovascular reactivity in relation to sleep–wake states. As expected, under LD conditions, REM and non-REM sleep were more prevalent during the light phase. In contrast, LL exposure disrupted the typical daily sleep-wake distribution, consistent with findings from rats previously exposed to dim light at night [35]. Furthermore, LL in our study increased sleep fragmentation and reduced the stability of circadian sleep rhythms, which may have adverse consequences for cardiovascular function [36].

Importantly, LL exposure disrupted non-REM sleep, reducing it during the light phase when it typically predominates, and fragmenting its distribution across the 24-h cycle. This pattern resembles consequences of sleep deprivation [37], when the restorative phases of sleep are compromised, even if total sleep time is not entirely abolished. REM sleep, conversely, was elevated under LL, particularly compared to the dark phase in LD cycles, indicating that both major sleep states were shifted away from their expected circadian timing. Together, these changes suggest that LL does not simply reduce total sleep but instead degrades sleep quality by redistributing and fragmenting both REM and non-REM phases.

Our results suggest that the disruption of circadian organisation by LL compromises the brain’s restorative sleep processes, potentially leading to increased fatigue and impaired recovery [38]. Non-REM sleep, characterised by delta waves, is crucial for brain regeneration and synaptic recovery in various animals [38]. A decline in delta wave activity in humans has been associated with cognitive impairment and neurodegenerative conditions, such as Alzheimer’s and Parkinson’s disease [39]. Moreover, non-REM activity correlates positively with the expression of CLOCK-regulated circadian genes, and this association is disrupted in neurodegenerative states such as tauopathies [39]. This relationship has been demonstrated in animal models and humans, including patients in the preclinical stages of Alzheimer’s disease [39]. Therefore, our data indicate that LL compromises sleep quality and circadian organisation rather than merely reducing sleep quantity, which may contribute to a broad spectrum of health impairments in nocturnal rats. Therefore, the pervasive disruption of LD cycles in recent decades [40] may contribute to the rising incidence of cardiovascular, neurodegenerative and metabolic diseases.

### Circadian and ultradian variability

We observed a reduction in mesor for cardiovascular variables. No such decline was found for locomotor activity and EEG-derived sleep parameters, indicating that the overall mean activity levels were unaffected despite the clear disruption of circadian structure. This finding also suggests that locomotor activity alone cannot fully explain the circadian variability of HR and BP [25]. In rats, locomotor activity is behaviourally masked by light [41], and cardiovascular changes following light exposure can reflect this response [42].

It is generally accepted that increasing light intensity progressively attenuates the circadian rhythmicity of locomotor activity and lengthens its free-running period [43]. Consistent with these findings, we observed a loss of circadian variability in locomotor activity. Previous studies have documented similar effects in both 8- and 33-week-old female mice exposed to LL [44]. Decreased amplitude of HR and locomotor activity was also observed after chronic restraint stress in Wistar-Kyoto and spontaneously hypertensive rats [45]. Our results confirm unchanged overall activity under LL in older female rats and raise questions about methodological factors and age-related research, in which infrared beam-break systems should be preferred over running wheels in evaluation of activity rhythms [44]. Moreover, this consideration also extends to methods how the data are analysed. Earlier studies have analysed harmonic ultradian oscillations [9, 13, 46], particularly useful for describing biphasic activity. However, since harmonic oscillations are integer multiples of the primary circadian rhythm, they arise as higher-frequency components. Therefore, here we applied spectral analysis to decompose the data into frequency components.

Rats exposed to LL showed reduced oscillatory power in the 3–5 h range, and a complete absence of ultradian variability in the 7–9 h range during LL4. For locomotor activity and BP, we observed that fewer LL-exposed rats had significant oscillations in the 7–13 h range. For HR, oscillations in the 7–9 h range decreased, while those in the 1–3 h range increased significantly. This increase could not be detected using harmonic oscillation analysis [24]. Our findings indicate that the observed reduction in more extended ultradian periods was offset by an increase in shorter periodic components. This suggests that the loss of circadian and ultradian variability in intervals dominated by endogenous control is likely due to a shift in oscillatory power towards shorter periods. This aligns with previous research on both normotensive and hypertensive male rats, which reported enhanced shorter ultradian oscillations after LL exposure [13]. Given that locomotor activity is known to rely on circadian variability, these results suggest that internal mechanisms underlie these alterations [24, 47]. The shift towards shorter ultradian periods after LL exposure may represent a physiological adaptation. Recent work suggests that ultradian rhythms in behaviour and metabolism are enhanced under negative energy balance or fasting-like conditions. In contrast, in positive energy balance, they can be masked by stronger circadian rhythms [11]. In this context, the LL-induced changes we observed could reflect an adaptive response to stress imposed by light exposure. This interpretation is supported by the known regulatory roles of the endocrine and autonomic nervous systems in influencing circadian and ultradian variability [10, 48]. However, the spectral profile of ultradian variability may differ depending on whether the signal originates from central [49, 50] or peripheral [1, 51] sources, as supported by mathematical and experimental models. Mouse studies show that changes in dopaminergic activity modulate the length of ultradian periods, supporting the hypothesis that dopamine regulates these rhythms independently of the master circadian pacemaker [49]. In the fly *Drosophila melanogaster*, specific neurons have been identified as ultradian oscillators at the central level that influence the circadian system and help maintain free-running rhythms [52].

Although the exact origins of ultradian rhythms and their interrelations with the circadian system remain under investigation, their importance for physiology is evident. Notably, a recent study elegantly demonstrated that in the mouse brain, 50-s noradrenaline pulses govern vascular dynamics and glymphatic flow. Disrupting this rhythm impairs brain clearance, which is essential for memory consolidation and the microstructure of sleep [53]. Based on our findings, we hypothesise that LL shifts the balance of the autonomic nervous system and may consequently diminish noradrenergic signalling. Such disruption, over time, may result not in hypertension in the absence of stress stimuli but rather in insufficient cerebral perfusion and increased susceptibility to neurodegenerative disease.

### Limitations

Our study was conducted with older female rats, without direct comparison with males or younger females. As noradrenaline levels are known to decline with age [54], this may limit the generalisation of our findings to other groups or males. The interpretation of our results should consider these sex- and age-related limitations.

## Conclusions

Exposure to LL profoundly disrupts the physiological state of female rats. Circadian and ultradian rhythms of HR, BP, locomotor activity, and sleep are weakened or lost, while sleep architecture becomes fragmented. Overall sleep duration does not increase, as expected for a nocturnal animal exposed to LL. Nevertheless, systolic BP progressively declines, pointing to impaired SCN output and altered central autonomic regulation rather than a direct consequence of reduced activity or increased sleep. Importantly, the reduction of daily variability increases the reactivity of cardiovascular parameters to stress. Together, our findings indicate that LL exposure induces a maladaptive state of persistent physiological activation. Subsequent disruption of cardiovascular and sleep–wake circadian rhythms may represent a pathway to increased cardiovascular and neurodegenerative risk, even in nocturnal animals.

## Supplementary information

SupplementaryTable S1

Supplementary Figure S1

Supplementary Figure S2

Supplementary Figure S3

Supplementary Figure S4

Supplementary data

## Data Availability

Data will be made available on request.
