# Supplementary material for: Constant light disrupts biological rhythms and worsens sleep quality but does not elevate blood pressure in female rats
Source: Hypertens Res. 2026 Feb 19;49(4):1349–60. doi: 10.1038/s41440-026-02579-8 (PMC13050649; doi:10.1038/s41440-026-02579-8)
Supplement: Supplementary file 1 — SupplementaryTable S1 [file 41440_2026_2579_MOESM1_ESM.pdf]

| Parameter | Comparison | Normality (p-val) | Method | p-val | Average ± SEM |
|---|---|---|---|---|---|
| SDNN | LD dark vs. LL | 0.170 | LMM | 0.141 | LD dark: 12.65 ± 0.75, LL1: 13.17 ± 0.72 , LL3: 13.86 ± 0.70 , LL4: 13.36 ± 0.43 |
| | LD light vs. LL | 0.876 | LMM | 0.313 | LD light: 13.70 ± 0.76, LL1: 13.17 ± 0.72 , LL3: 13.86 ± 0.70 , LL4: 13.36 ± 0.43 |
| | LD dark vs. light | 0.834 | Paired t-test | 0.224 | dark: 12.65 ± 0.75, light: 13.70 ± 0.76 |
| RMSSD | LD dark vs. LL | 0.766 | LMM | 0.064 | LD dark: 4.41 ± 0.12, LL1: 4.90 ± 0.52 , LL3: 6.14 ± 1.06 , LL4: 6.03 ± 0.98 |
| | LD light vs. LL | 0.952 | LMM | 0.056 | LD light: 4.80 ± 0.32, LL1: 4.90 ± 0.52 , LL3: 6.14 ± 1.06 , LL4: 6.03 ± 0.98 |
| | LD dark vs. light | 0.568 | Paired t-test | 0.200 | dark: 4.41 ± 0.12, light: 4.80 ± 0.32 |
| LF power | LD dark vs. LL | 0.361 | LMM | 0.761 | LD dark: 1.44 ± 0.07, LL1: 1.47 ± 0.17, LL3: 1.63 ± 0.30, LL4: 1.64 ± 0.34 (values $x10^{-4}$) |
| | LD light vs. LL | 0.143 | LMM | 0.438 | LD light: 1.34 ± 0.12, LL1: 1.47 ± 0.17, LL3: 1.63 ± 0.30, LL4: 1.64 ± 0.34 (values $x10^{-4}$) |
| | LD dark vs. light | 0.131 | Paired t-test | 0.379 | dark: 1.44 ± 0.07, light: 1.34 ± 0.12 (values $x10^{-4}$) |
| HF power | LD dark vs. LL | 0.371 | LMM | 0.064 | LD dark: 2.14 ± 0.12, LL1: 2.31 ± 0.30, LL3: 3.81 ± 0.96, LL4: 4.02 ± 1.12 (values $x10^{-4}$) |
| | LD light vs. LL | 0.384 | LMM | 0.075 | LD light: 2.19 ± 0.08, LL1: 2.31 ± 0.30, LL3: 3.81 ± 0.96, LL4: 4.02 ± 1.12 (values $x10^{-4}$) |
| | LD dark vs. light | 0.338 | Paired t-test | 0.393 | dark: 2.14 ± 0.12, light: 2.19 ± 0.08 (values $x10^{-4}$) |
| LF nu | LD dark vs. LL | 0.462 | LMM | 0.000 | LD dark: 0.440 ± 0.007 , LL1: 0.435 ± 0.010 , LL3: 0.369 ± 0.021 , LL4: 0.352 ± 0.016 |
| | LD light vs. LL | 0.579 | LMM | 0.011 | LD light: 0.420 ± 0.012 , LL1: 0.435 ± 0.010 , LL3: 0.369 ± 0.021 , LL4: 0.352 ± 0.016 |
| | LD dark vs. light | 0.856 | Paired t-test | 0.254 | dark: 0.440 ± 0.007 , light: 0.420 ± 0.012 |
| HF nu | LD dark vs. LL | 0.462 | LMM | 0.000 | LD dark: 0.560 ± 0.007 , LL1: 0.565 ± 0.010 , LL3: 0.631 ± 0.021 , LL4: 0.648 ± 0.016 |
| | LD light vs. LL | 0.579 | LMM | 0.011 | LD light: 0.580 ± 0.012 , LL1: 0.565 ± 0.010 , LL3: 0.631 ± 0.021 , LL4: 0.648 ± 0.016 |
| | LD dark vs. light | 0.856 | Paired t-test | 0.254 | dark: 0.560 ± 0.007 , light: 0.580 ± 0.012 |
| LF/HF | LD dark vs. LL | 0.426 | LMM | 0.001 | LD dark: 0.881 ± 0.029 , LL1: 0.865 ± 0.034 , LL3: 0.664 ± 0.067 , LL4: 0.613 ± 0.048 |
| | LD light vs. LL | 0.666 | LMM | 0.009 | LD light: 0.821 ± 0.042 , LL1: 0.865 ± 0.034 , LL3: 0.664 ± 0.067 , LL4: 0.613 ± 0.048 |
| | LD dark vs. light | 0.970 | Paired t-test | 0.283 | dark: 0.881 ± 0.029 , light: 0.821 ± 0.042 |
| BRS | LD dark vs. LL | 0.026 | Friedman test | 0.017 | LD dark: 3.91 ± 0.14 , LL1: 3.76 ± 0.21 , LL3: 5.11 ± 0.81 , LL4: 5.45 ± 1.19 |
| | LD light vs. LL | 0.015 | Friedman test | 0.017 | LD light: 3.78 ± 0.19 , LL1: 3.76 ± 0.21 , LL3: 5.11 ± 0.81 , LL4: 5.45 ± 1.19 |
| | LD dark vs. light | 0.599 | Paired t-test | 0.143 | dark: 3.91 ± 0.14 , light: 3.78 ± 0.19 |
