# Supplementary figures and images for: Constant light disrupts biological rhythms and worsens sleep quality but does not elevate blood pressure in female rats
Source: Hypertens Res. 2026 Feb 19;49(4):1349–60. doi: 10.1038/s41440-026-02579-8 (PMC13050649; doi:10.1038/s41440-026-02579-8)

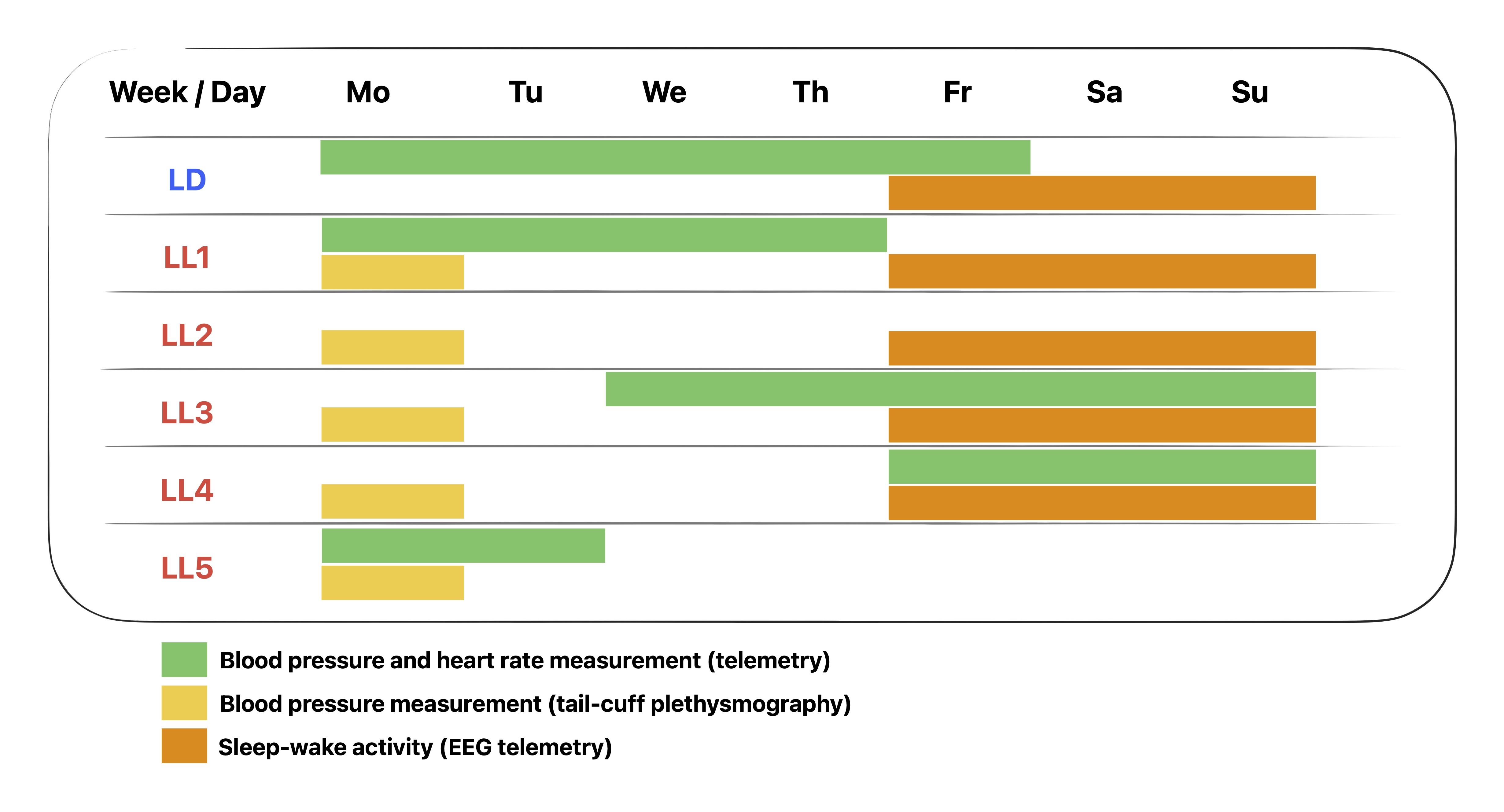

Supplement: Supplementary file 2 — Supplementary Figure S1 [file 41440_2026_2579_MOESM2_ESM.jpg]

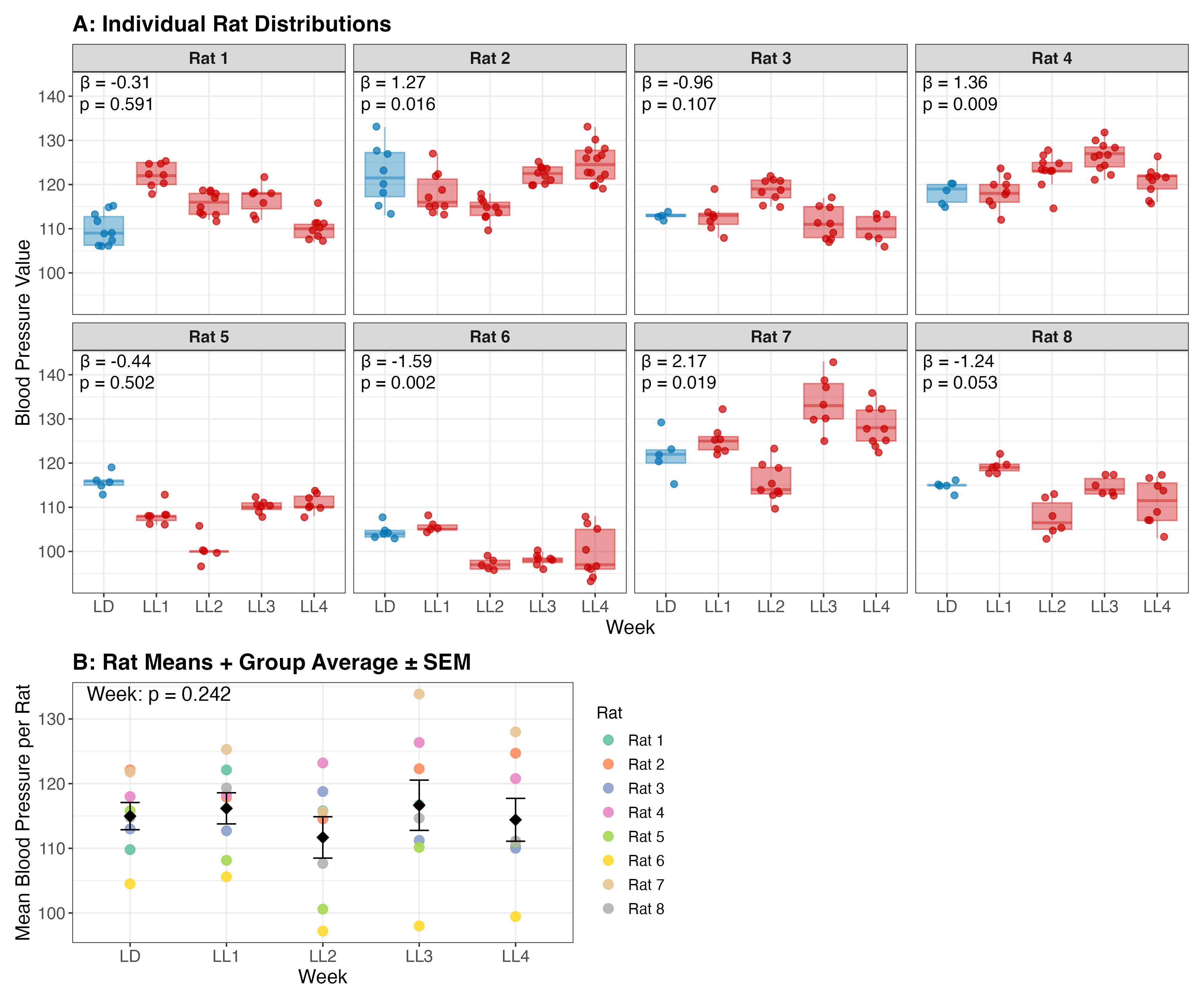

Supplement: Supplementary file 3 — Supplementary Figure S2 [file 41440_2026_2579_MOESM3_ESM.jpg]

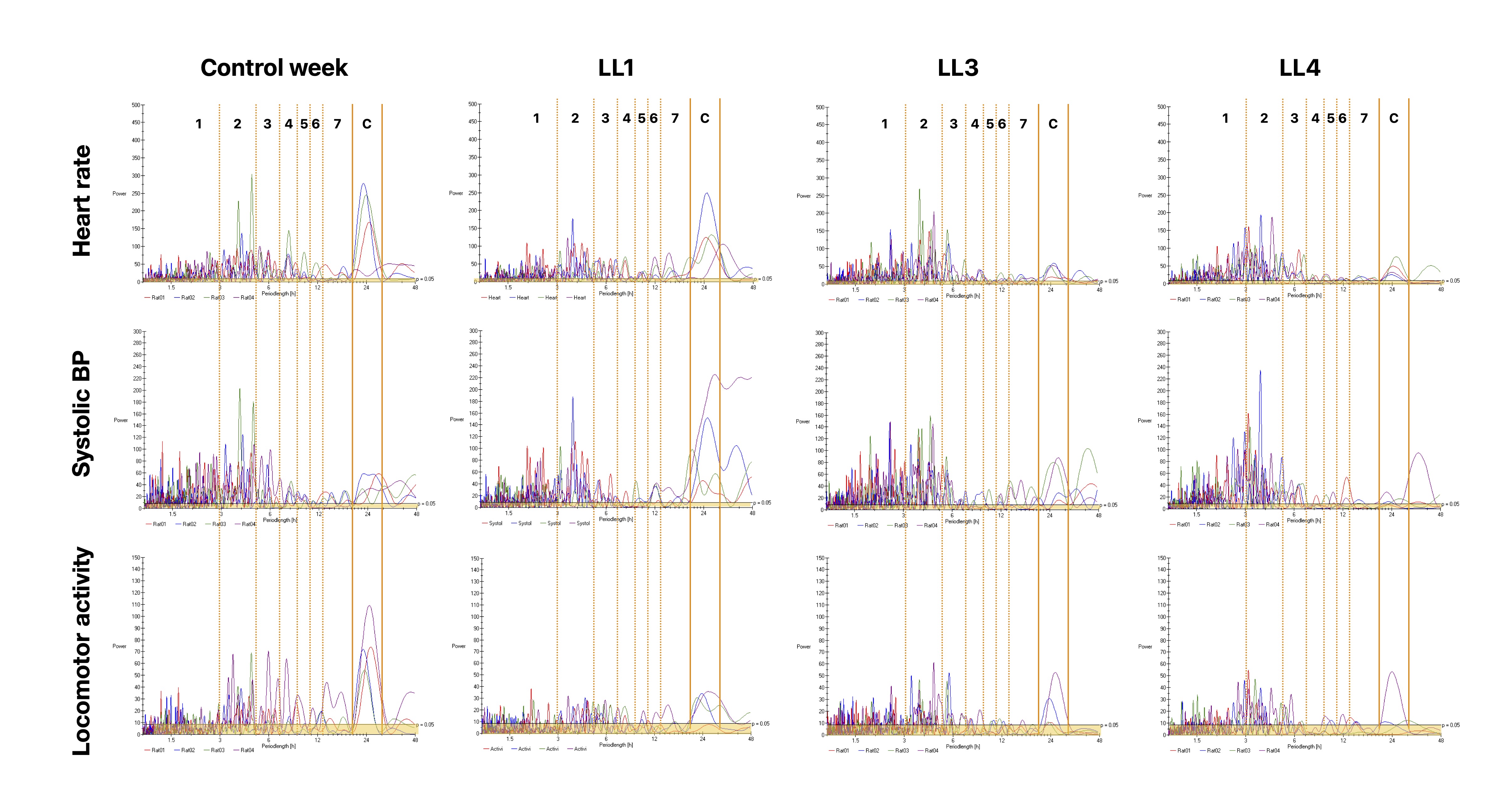

Supplement: Supplementary file 4 — Supplementary Figure S3 [file 41440_2026_2579_MOESM4_ESM.jpg]

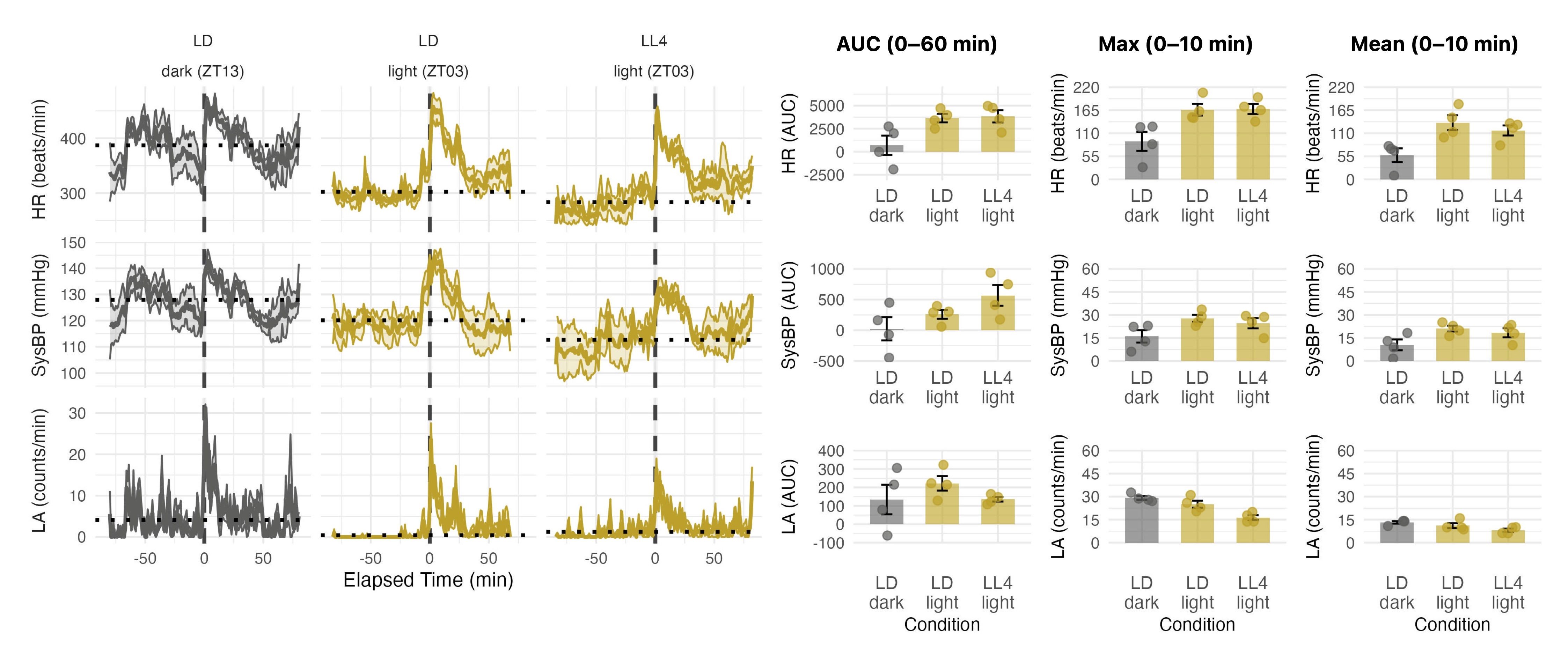

Supplement: Supplementary file 5 — Supplementary Figure S4 [file 41440_2026_2579_MOESM5_ESM.jpeg]
