# Supplementary material for: Constant light disrupts biological rhythms and worsens sleep quality but does not elevate blood pressure in female rats
Source: Hypertens Res. 2026 Feb 19;49(4):1349–60. doi: 10.1038/s41440-026-02579-8 (PMC13050649; doi:10.1038/s41440-026-02579-8)
Supplement: Supplementary file 6 — Supplementary data [file 41440_2026_2579_MOESM6_ESM.docx]

# Supplementary data

**Figure S1: Experimental timeline and measurement schedule.** The figure illustrates the weekly schedule of experimental procedures across the control (LD) and constant light (LL) weeks. Green bars indicate periods of continuous blood pressure and heart rate monitoring by telemetry. Yellow blocks mark the days when blood pressure was assessed using tail-cuff plethysmography. Orange bars represent sleep-wake activity measured by electroencephalography.

**Figure S2: Individual and group-level changes in systolic blood pressure measured by tail-cuff plethysmography across four weeks of constant light exposure.** (A) Box plots showing weekly distributions of systolic blood pressure values in individual rats (n = 8) during the light–dark (LD) week (control) and four subsequent weeks of constant light (LL1–LL4). Linear regression slopes (β) and p-values for each rat are reported to indicate direction and significance of changes over time. (B) Group summary plot showing mean systolic blood pressure per rat per week (dots), along with group average ± SEM (black triangles and error bars).

**Figure S3: Lomb–Scargle periodograms of cardiovascular and activity parameters under light-dark cycle and constant light exposure.** Periodograms show the spectral power distribution across periods ranging from ultradian to circadian ranges (x-axis: period in hours) for heart rate, systolic blood pressure (Systolic BP), and locomotor activity in individual rats (colour-coded) during the light–dark (LD) week (control) and three of four subsequent constant light weeks (LL1, LL3, LL4). Vertical dashed orange lines mark predefined ultradian frequency ranges (1–12 h), and thick dashed orange lines highlight the circadian range (~24 h). 1–7, ultradian intervals 1–3 h, 3–5 h, 5–7 h, 7–9 h, 9–11 h, 11–13 h and 13–20 h; C, circadian interval (20–28 h). Time intervals with spectral power rising above the yellow horizontal line indicate statistically significant rhythms (p < 0.05).

**Figure S4: Cardiovascular and behavioural responses to acute shaking stress under different lighting conditions.** The left panels show the time course of heart rate (HR), systolic blood pressure (SysBP), and locomotor activity (LA) in response to a shaking stimulus (time 0, dashed line) during the dark and light phases of a standard light-dark (LD) cycle and week 4 of constant light (LL4). Middle panels represent the area under the curve (AUC) for each parameter over a 60-min, poststimulus window. Right panels show the maximal and the mean changes in the 10 min poststimulus. Data are shown as means ± SEM; dots indicate individual rats.

**Table S1: Effect of constant light exposure on heart rate variability (HRV) indices and baroreflex sensitivity (BRS).** Data are presented as mean ± SEM. Comparisons were performed between LD (12 h light/12 h dark) and LL (constant light) conditions, separately for the light and dark phases of LD. Statistical analysis was carried out using linear mixed-effects models (LMM) or paired t-tests. For BRS, a non-parametric Friedman test was applied (overall p = 0.017). Units for low-frequency (LF) and high-frequency (HF) power are ×10⁻⁴, for LF and HF nu, the values are in normalised units.
